# Supplementary figures and images for: Using unstructured crowd-sourced data to evaluate urban tolerance of terrestrial native animal species within a California Mega-City
Source: PLoS One. 2024 May 29;19(5):e0295476. doi: 10.1371/journal.pone.0295476 (PMC11135677; doi:10.1371/journal.pone.0295476)

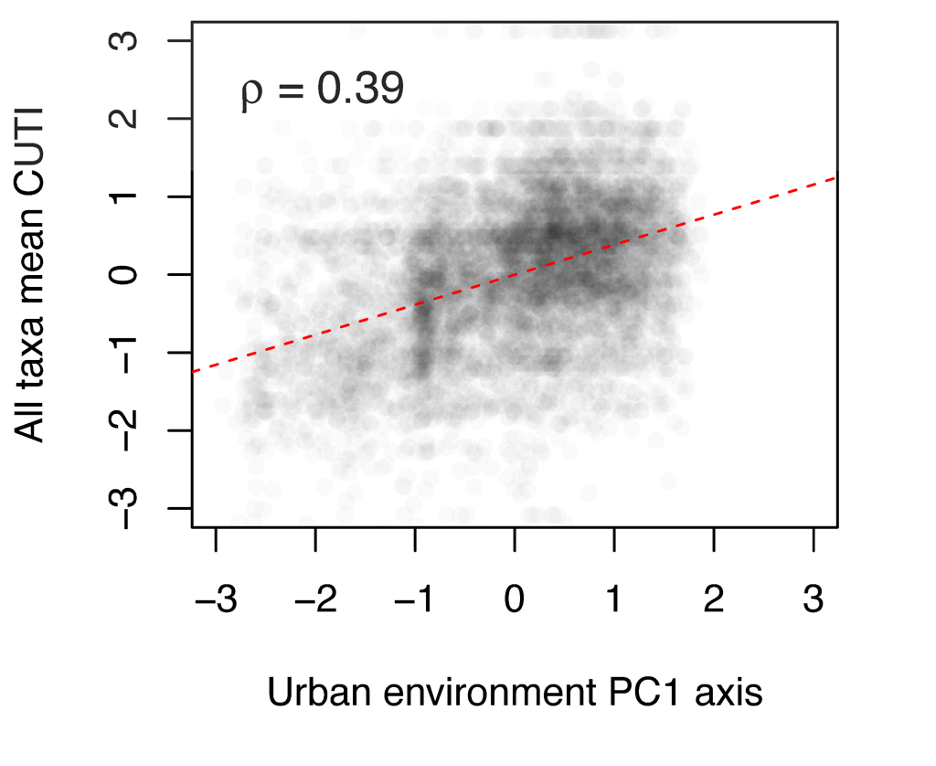

Supplement: S1 Fig — (TIF) [file pone.0295476.s001.tif]
