# Supplementary material for: Using unstructured crowd-sourced data to evaluate urban tolerance of terrestrial native animal species within a California Mega-City
Source: PLoS One. 2024 May 29;19(5):e0295476. doi: 10.1371/journal.pone.0295476 (PMC11135677; doi:10.1371/journal.pone.0295476)
Supplement: S1 Table — (DOCX) [file pone.0295476.s002.docx]

**Supplementary Table 1.** Table detailing the loadings and variance explained for the composite urban intensity layer separated by contributing layers and PC axes.

| PC Axis | **Spatial layer loadings** | | | **Percentage of variance explained** | **Cumulative percentage of variance** |
| --- | --- | --- | --- | --- | --- |
|  | *Impervious surfaces* | *Night-time lights* | *Noise pollution* |  |  |
| PC1 | 0.560 | 0.601 | 0.570 | 86% | 86% |
| PC2 | 0.752 | -0.080 | -0.654 | 10% | 96% |
| PC3 | 0.347 | -0.795 | 0.497 | 4% | 100% |
