# Supplementary material for: Using unstructured crowd-sourced data to evaluate urban tolerance of terrestrial native animal species within a California Mega-City
Source: PLoS One. 2024 May 29;19(5):e0295476. doi: 10.1371/journal.pone.0295476 (PMC11135677; doi:10.1371/journal.pone.0295476)
Supplement: Attachment — Submitted filename: ResponseToReviewers_PLOS_Curti.docx [file pone.0295476.s004.docx]

Response to Reviewers

**Summary: We thank the editor and reviewers for their feedback on our manuscript. We have addressed each concern, specifically adding clarification and additional details to the manuscript, where necessary. Responses to comments are presented below, in bold.**

## Academic Editor (Journal Requirements)

1. Please ensure that your manuscript meets PLOS ONE's style requirements, including those for file naming.

**We have worked to follow these style conventions.**

2. Please note that PLOS ONE has specific guidelines on code sharing for submissions in which author-generated code underpins the findings in the manuscript. In these cases, all author-generated code must be made available without restrictions upon publication of the work. Please review our guidelines and ensure that your code is shared in a way that follows best practice and facilitates reproducibility and reuse.

**We have made our code publicly available on Zenodo and the embargo will be lifted upon publication.**

3. When completing the data availability statement of the submission form, you indicated that you will make your data available on acceptance. We strongly recommend all authors decide on a data sharing plan before acceptance, as the process can be lengthy and hold up publication timelines. Please note that, though access restrictions are acceptable now, your entire data will need to be made freely accessible if your manuscript is accepted for publication. This policy applies to all data except where public deposition would breach compliance with the protocol approved by your research ethics board. If you are unable to adhere to our open data policy, please kindly revise your statement to explain your reasoning and we will seek the editor's input on an exemption. Please be assured that, once you have provided your new statement, the assessment of your exemption will not hold up the peer review process.

**We have uploaded a new document to the supplement titled, “RawData_PLOS_Curti.csv” which contains a link to each unfiltered iNaturalist observation used in this study. This format and method adheres to the guidelines set forth by iNaturalist for citing and providing access to their data.**

4. We note that Figures 1,3 and 4 in your submission contain [map/satellite] images which may be copyrighted. All PLOS content is published under the Creative Commons Attribution License (CC BY 4.0), which means that the manuscript, images, and Supporting Information files will be freely available online, and any third party is permitted to access, download, copy, distribute, and use these materials in any way, even commercially, with proper attribution. For these reasons, we cannot publish previously copyrighted maps or satellite images created using proprietary data, such as Google software (Google Maps, Street View, and Earth). We require you to either (1) present written permission from the copyright holder to publish these figures specifically under the CC BY 4.0 license, or (2) remove the figures from your submission:

**We have created a new document describing figure and spatial data permissions and uploaded it as “other” in our updated submission.**

5. Please upload a copy of Supporting Information (Supplementary Table 1 and 2 and Supplementary Figure 1) which you refer to in your text on page 21.

**We included supplementary information in the original submission via a private reviewer link to the Zenodo upload. I’ve included this link again below in case you were previously unable to access.

Link to Zenodo:**
https://zenodo.org/records/10807319?token=eyJhbGciOiJIUzUxMiJ9.eyJpZCI6IjQ3NTdkY2ZlLWQxYjAtNDFiYi1hMmNmLWVhZDk3ZWU5MzNiZCIsImRhdGEiOnt9LCJyYW5kb20iOiIyOTM4NzU3ZDJjMGYyYjQyYTU4YTVjOWZkNWI1ZDdhYiJ9.itICB4EXI1RumytIVV4jEOmrOfKZlsjsdbtECO68rrEm7TH6RymfXWZszkraI1SK6p6sTL_S7QIKmUEV9E8TIQ

**If the editor would prefer Supporting Information in another way, or if we are misunderstanding PLOS ONE’s policies, we will be happy to conform in whatever way necessary.**

6. Please review your reference list to ensure that it is complete and correct. If you have cited papers that have been retracted, please include the rationale for doing so in the manuscript text, or remove these references and replace them with relevant current references. Any changes to the reference list should be mentioned in the rebuttal letter that accompanies your revised manuscript. If you need to cite a retracted article, indicate the article’s retracted status in the References list and also include a citation and full reference for the retraction notice.

**We have reviewed the reference list.**

## Academic Editor (additional comments)

The manuscript has several problems regarding formatting to PLOS ONE. Please review the Submission Guidelines and follow the instructions regarding this aspect in every section (below).
General: Manuscript text should be double-spaced.

**We have made the manuscript double spaced.**

You have to use a numerical system for citations. See recent PLOS ONE papers. Examples:
[2] note the square brackets. For a single ref.
[4, 5]…….[3, 6] note the space after the comma. For two refs.
[4 ̶ 6]…..use a long dash, not hyphen, without space. For 3 or more refs.

**We now use a numbered reference system.**

Abstract: Well done. Use “Abstract” (only the first letter in capital).
Introduction: Use “Introduction”. Only the first in capital. The same for other top section titles.
Very good content and length. Objectives were well explained.
Methods: Use “Methods”
And “Study area” in bold, not italics.

**We have completed these edits.**

Study Area: It would be good to provide some brief information, in a few sentences, about the environment of this region, with references (e.g. climate, terrain, original vegetation, landscapes….). You will have an international readership.

**We have provided a brief section on Southern California climate, vegetation, and habitat types starting on line 151.**

Line 164. Subtitle. In bold, not italics.

**We have changed this throughout the revised manuscript.**

Line 184. S1 Table. I could not find its link. Have you added this to the submission ?

**Please see the above link that was also included in the original submission.**

Line 187. Use “Fig 1” (without the dot).

**We have changed this throughout the revised manuscript.**

Line 196. In bold, not italics. The same for other subtitles.

**We have changed this throughout the revised manuscript.**

Lines 202-205. Should these family names be in italics ? I think not. Please check this. Text and tables.

**We have changed throughout the manuscript. Our formatting is now in line with ICZN best practices (**[**https://www.iczn.org/assets/6ecc2ac99f/best-practice.pdf**](https://www.iczn.org/assets/6ecc2ac99f/best-practice.pdf)**).**

Results: There is no need for bold when citing figures and tables. Only in captions and titles.

**We have changed this throughout the revised manuscript.**

Line 326. Use “Fig. 1, Fig 2, S2 Table). Have you included this table in the manuscript ? There is no link.

**Please see the above link that was also included in the original submission.**

Table 1. You have to provide one or more words that comprise the top names of columns 1-3. Such as “Taxa information” ?

**We have used “Taxa Information” as you suggest for the column header.**

Line 363. Have you included S1 Fig in the manuscript ? Use “S1 Fig”.

**Yes, this figure is first referenced on line 521.**

Linne 365. Something was cut. Please complete.

**Thank you for noticing this. This was an error and it has been fixed in the revised manuscript.**

Discussion: You could consider adding subtitles along the discussion to ease the reading.

**Thank you, this is a good idea. I have added subtitles to the discussion to improve readability.**

Line 427. Maybe start a new paragraph.

**Thank you for your suggestion. Since this is still specific to the paragraph about butterfly conservation, we propose that this is best kept as a single paragraph. We have clarified this in text to make sure it is clear that the recommendations are for butterflies.**

Your discussion misses an ecological section….try to add 1-2 paragraphs to discuss factors that could influence the positive, neutral and negative responses of different animals to urbanization. Excessive noise/light, lack of resources, presence of gardens, urban parks. Given attention to the three components of urbanization.

**We have added two paragraphs starting on line 456 discussing additional intrinsic and extrinsic factors that can influence the responses of animals to urbanization.**

Supplementary material
Apparently, your submission does not have it.
You have to add them when submitting the corrected version.
Here in the end of the ms, you have to add the titles of S1 tables and captions of S1 figures.

**Please see the above link that was also included in the original submission. Also, titles and captions of supplementary tables and figures were included in the original submission and are retained in the revised manuscript starting around line 1706.**

References: Please check again Submission Guidelines and recent PLOS ONE papers for correct formatting. Some major problems:
This alphabetical order is not acceptable. You have to list the references according to the order in which they appear along the text.
You have to add DOI for all references that bring this information. Format: <https://doi.org/10...>..
For refs with journals, It should be: Journal. Year; volume(issue):pages. Months are not necessary.
Titles of journals should be abbreviated.
For several refs, you placed the first letter of words in capital; it should not occur, except for proper names, etc…
Check also books, chapters and webpages, etc…..

**We have reformatted our references so that they are in the proper format.**

## Reviewer 1

Summary: This manuscript details a study evaluating the urban tolerance of native animal species in Los Angeles, California, using occurrence data from iNaturalist. Using a composite urbanization rank, 510 native animal species were evaluated for their urbanization associations and animal communities were evaluated for their urban tolerance across LA. The authors found that urbanization responses were taxa-specific, with snails and slugs were most positively associated with urbanization and butterflies and moths were least. This manuscript is well written, comprehensive in its scope, and has a strong analytical design, especially regarding the use of crowd-sourced data. Few studies take such a taxa-inclusive approach to describing patterns in community composition and occurrence across urbanization gradients, which will make this paper stand out in the literature. I commend the authors on such a strong submission. Below are a few minor points to address for clarity.

**We thank the reviewer for their efforts in evaluating our manuscript and for their gracious complements.**

Title: May want to indicate that terrestrial native animal species were the focus of this paper.

**To reflect that this paper focuses on animal species, we have changed the title from, “Using Unstructured Crowd-sourced Data to Evaluate Urban Tolerance of Terrestrial Native Species within a California Mega-City”, to “Using Unstructured Crowd-sourced Data to Evaluate Urban Tolerance of Terrestrial Native Animal Species within a California Mega-City”**

Line 36: The statement “e.g., mammals are not the same as birds” seems unnecessary to specify here.

**We have removed this statement from the revised manuscript.**

Lines 60–76: Regarding taxa-specific responses to urbanization, I would suggest reading Hahs et al. 2023 “Urbanisation generates multiple trait syndromes for terrestrial animal taxa worldwide” in Nature Communications. Many of the same taxonomic groups were sampled in this manuscript and Hahs et al. regarding their functional traits and urbanization associations. Right now, this paragraph and the next heavily focus on birds and their associations with plants in cities. While birds have historically been one of the major animal taxonomic groups studied in the urban biodiversity literature, there were 11 more taxonomic groups surveyed in this paper that are worth mentioning here.

**We appreciate this suggestion. We have added additional examples of how factors of the local environment and sampling resolution can impact the relationship between urbanization and biodiversity. Notably, we drew from examples in mammals and invertebrates to make this section more representative of the different taxonomic groupings that are present in this study. We believe that this addresses the concern.**

Line 62: Rega-Brodsky et al. 2022 did not report an association between urbanization and loss of plant diversity, only that the bulk of urban biodiversity literature sampled plants. If anything, publications resulting from that research group indicated that cities were able to support diverse plant communities (Aronson et al. 2014 Proc. R. Soc. B).

**We have broadened the language around line 60 to better illustrate our intended point that urban biodiversity research has primarily been conducted using a limited subsect of taxa (i.e., not implying the direction of the response).**

Figure 1: The boundary line for the City of LA would be helpful to use here to standardize with the remaining maps (Figs 3 & 4). Are major roads necessary to indicate here?

**We have added the outline of the city of Los Angeles to this map. After experimentation with multiple versions, we believe that the addition of major roadways helps make the spatial variation of urban intensity more interpretable, especially to readers who are not familiar with the geography of the Los Angeles region. Therefore, we have retained roadways in the updated version of the map but changed the color so as to highlight the city boundary.**

Figure 2: Since snails/slugs were the most urban tolerant taxonomic group, might it be beneficial to include at least one as an example here? Why were these three species selected beyond their urbanization ranks?

**We wanted to select examples that 1) typified the full variation of UAI relationships (i.e., urban associated, urban avoiding, and no urban relationship), 2) had lots of data supporting those relationships, 3) showed diversity across taxa studied, and 4) would be species that are recognizable to both a general and local readership (i.e., charismatic ambassadors matter). Therefore, after consideration, we have decided to retain the three species depicted.**

Line 323: Table 1 indicates that 487 species remained following the filters applied to the query. However, this statement states that 967 species remained following the filtered query and of those, 563 occurred more than once, 510 had UAI calculated, and 408 had UAI and occurred more than once. Where does n=487 fit in?

**This was an error and we appreciate the reviewer’s sharp eyes. The correct number for the table is 967, not 487. We have corrected table 1.**

Table 1: For those unfamiliar with iNat, please indicate in the table title what ‘iNat Taxa ID’ represents (or can that information be moved to Supplemental Material?). I would also remind readers that these were specifically only native species of these taxonomic groups (i.e. were the non-native species removed pre- or post-filter?).

**We have attempted to clarify this in the header for Table 1. We also now refer in the Table legend that filtered results exclude non-native species and refer readers to that section of the Methods.**

Table 2: This table might be better presented as a figure, with mean and range values indicated on a UAI gradient. Perhaps the UAI can be on the x-axis (centered at zero) with each taxonomic group on the y-axis, with the mean as a point and min/max UAI represented with lines radiating from the point (or perhaps smaller dots representing each species within the taxonomic group). This would provide a better visual of where each group lands regarding their urban tolerance/intolerance. If retaining these data as a table, ranges should at least be provided for each taxonomic group.

**We appreciate this suggestion and have explored the options for changing the table to a figure. Given the varying numbers of species per taxonomic group, it is hard to visualize the individual (species) data points in an effective way across groups. Thus, we have decided to stick with a table and have provided ranges in parentheses within the table.**

Line 358: Please define ‘IUM’.

**This is a typo and was changed to “CUTI” in revision.**

Line 365: This final sentence is incomplete.

**This sentence was in error and has been removed.**

Discussion: It might be worth pointing out that some of the taxonomic groups studied were at the Family level, while others were at the level of taxonomic Order, which may obscure subtleties in urbanization responses. Even within one taxonomic Family/Order, there are large differences in urbanization response across native species and their populations (See Weiss et al. 2023 Ecology “Effect of species-level trait variation on urban exploitation in mammals”).

**We have added discussion of this around line 819.**

## Reviewer 2

In this study the authors present a methodological approach on how to make use of open access opportunistic and citizen-science-based data to explore the relationship between the urban environment and biodiversity. The proposed methodology sounds reasoned and repeatable and gives insight into the association (or avoidance) of various species with the urban environment. Below the authors can find some comments which I hope will help them to make some points of the manuscript clearer.

**We thank the reviewer for their review of our manuscript.**

GENERAL COMMENTS: The discussion focuses a lot on the use of iNaturalist as a source of biodiversity data and tool for environmental city planning. However, although the statistical analysis included several taxa, the discussion only mentions the most tolerant and intolerant species (snails and slugs and some butterflies respectively), whereas it does not comment on the responses of the rest of the taxonomic groups. I think a paragraph describing the trends of the rest of the taxonomic groups could be added.

**The handling editor and the other reviewer similarly commented on this and we have added two additional paragraphs starting at line 506 that discuss intrinsic and extrinsic factors that can explain our results. In these paragraphs, we discuss other relationships of our taxonomic groupings to levels of urbanness including insects and birds**

Although the end of the introduction section is rather untypical, as it summarizes methodology and includes some conclusions and suggestions, I would not disagree with this format if this was the authors’ choice.

**We have kept the introduction as is because we believe it effectively sets up the motivation for the work.**

The authors use the term ‘community science’ rather than ‘citizen science’ which is more commonly used in biodiversity studies. Similarly, they use the term ‘crowd-sourced data’. I suggest they also add “citizen science” either to the keywords or to the text to facilitate possible future search (e.g. for meta-analyses and review articles).

**The reviewer is right to identify that the terminology here is a bit confusing as the term ‘citizen science’ has become politicized in some areas and as different countries and disciplines use different terms to mean the same thing. We appreciate the creative solution of using keywords as a crosswalk and we have added ‘citizen science’ to the keywords for the paper.**

I suggest the authors avoid the word ‘urbanness’ and use more formal ones such as ‘urban intensity’, which is more widely used.

**We have changed “urbanness” to “urban intensity” throughout the revised manuscript.**

The family and order/class names should not be italicized according to the Zoological nomenclature (<https://www.iczn.org/>), only the species and the genera.

**We have reflected this change in the text.**

There are some abbreviations in the text which are not explained, e.g. PM2.5 (line 173), PM1.2b (line 136, 297), IUM (line 358).

**We have removed IUM because this was a typo and have provided definitions for the others terms where they are first presented in the text.**

SPECIFIC COMMENTS
L. 21 – Abstract: ‘city’ not capitalized.

**We have corrected this in the revised manuscript.**

L. 137: perhaps ‘repeated’ instead of ‘repeat’?

**We have corrected this in the revised manuscript.**

L. 174: What is the reference for PM2.5, Average Annual Traffic Volume, and Population Density? Although not used in the analysis due to collinearity, I suggest the authors add a reference for future use by other studies.

**We have added in citations for these data layers for future reference.**

L. 198: Perhaps ‘selected’ instead of ‘select’?

**We have corrected this in the revised manuscript.**

L. 225-228: How many scientists from the Expert Council were asked to evaluate the data used? Did they evaluate all species included in this study (from the 12 taxonomic groups) one by one? Did all experts examine the same set of species?

**We have clarified this in text around line 244.**

L. 237-246: Have the authors considered the issue/effect of “iconic” and “charismatic” species that are more often recorded by citizens and amateurs? And alternatively, of species that are nearly never recorded because they are very common and abundant and observers think it is not worth noting them down?

**This is an interesting point. We agree that both of these things could be true (i.e.,, ‘charismatic’ species are recorded more often and ‘very common’ species are greatly under-recorded). If we consider charismatic species to be those with especially large sizes and bright colors, this seems quite plausible; for example, Callaghan et al. (2021) found that larger bird species were overrepresented in iNaturalist, although not more colorful species. It is also the case that those observations are more likely to be identified to a usable taxonomic level for the purposes of this study. With regards to common species reporting, we doubt that in iNaturalist there is too much bias against ‘very common’ species because that effect would likely vary greatly by user experience (and in citizen science, the majority of users are ‘new’), and any saturation of novelty is dependent on geographic familiarity (e.g., an iNaturalist user visiting Los Angeles from Seattle might be delighted to capture all the ‘common’ species here). Callaghan et al. (2021) reported “moderate” overrepresentation of common species in birds in iNaturalist.**

**Nevertheless, regardless of any true bias in reporting frequency, we take steps to control observer bias by 1) performing a spatio-temporal filter step on all records, whereby any one species in any one grid cell can have a maximum of one observation per year, and 2) controlling for varying effort of observers for different taxonomic groups by creating an effort layer for each taxonomic group within each grid cell. Using these methods, an observation of any species within a taxonomic group and within a given grid cell receives a value of one if it is detected in a given year (i.e., if five lepidopterans are observed within a grid cell between 2011-2021, that grid cell receives a score of 5/11) , and this value represents a maximum number of total observations for that taxonomic group when scoring UAI for a given species. In this way, we can control for uneven effort across taxonomic groups that lead to some species or groups being overrepresented in our data set.**

**In summary, since the majority of our inference is based on spatial comparisons of species occurrence (and our metric of urban infiltration), this inference would only be biased if (1) the biases suggested above are true with iNaturalist data in our study region, and (2) if the biases suggested above themselves are spatially biased (i.e., bias is non-randomly distributed across space). While it is possible that these biases may be true (i.e., over- or under-representation), we do not expect them to be spatially biased.**

**However, we recognize that it is important to caveat that our measure is, itself, imperfect due to the nature of the data. We have included a discussion of the potential impact of these biases on our dataset starting around line 535.**

L. 262: I think it would help to note here what is the number of 0.25 x 0.25 mile grid cells that were assessed.

**This information is already presented in the first sentence of the results on line 389. Therefore, we have not included that again here.**

L. 282: Thus, does this mean that each species occurred in at least 25 grid cells across the study region in all of the 11 sampling years?

**Yes, if any given species had at least 25 records after spatiotemporal thinning across all of the grid cells across the study region, they were included.**

L. 289-292: I think it would be helpful to add some more detail like “Species’ UAI scores are the logit-linear slope of the trend line ()” which is mentioned in line 339.

**We have used the suggested wording from line 339 as you indicate here in the methods at line 311.**

L. 298: Perhaps “of” instead of “or”?

**Thanks for catching this typo. This should actually be “for”, as in “To calculate a taxonomic group-specific CUTI for each grid cell in Los Angeles …”**

L. 358: It is not defined what “IUM” is.

**This is a typo; we have changed it to “CUTI”.**

L. 365: It seems that the sentence is incomplete.

**This sentence was in error and has been removed.**

L. 373: I assume the authors refer to Figure 3 instead of 2.

**Correct, this was a typo and should be “Figure 3”.**

L. 380: “a” is redundant.

**We have reflected this change in the text.**

L. 390: I think it would be helpful here to mention again what are the parameters measured for quantifying urban intensity (i.e. light pollution, impervious surfaces, noise pollution). Also, I suggest replacing ‘urbanness’ with ‘urban intensity’ (also l. 392).

**We have reflected both of these suggestions throughout the revised text.**

Figures 3 & 4: Please make sure that the maps are colour-blind friendly.

**I appreciate this suggestion. The lead author of the paper has mild color blindness (Deuteranomaly) and specifically chose the map symbology to account for this.**

Table 3: Do the authors think that it would be more informative to also add the CUTI values for Los Angeles city, together with the CUTI values for the entire study area?

**Table 3 depicts only CUTI values for the city of Los Angeles, since we did not calculate CUTI elsewhere. The study uses the city as a case study for how other cities can focus on biodiversity within their city limits. Therefore we think it would be a clearer narrative to leave these values as just Los Angeles.**
